# Distance between honey bee *Apis mellifera* colonies regulates populations of *Varroa destructor* at a landscape scale

**DOI:** 10.1007/s13592-016-0443-9

**Published:** 2016-05-02

**Authors:** Maxcy P. Nolan, Keith S. Delaplane

**Affiliations:** Department of Entomology, University of Georgia, 413 Biological Sciences Building, Athens, GA 30602 USA

**Keywords:** *Apis mellifera*, parasite transmission, host-parasite interaction, colony collapse

## Abstract

Inter-colony distance of *Apis mellifera* significantly affects colony numbers of the parasitic mite *Varroa destructor*. We set up 15 apiaries, each consisting of two colonies. Each apiary pair was assigned an inter-colony distance of 0, 10, or 100 m. Colonies were rendered nearly mite-free, then one colony in each pair was seeded with 300 female mites (mite-donor colony), while the other remained uninoculated (mite-recipient colony). After 4 months of monitoring, a whole-model analysis showed that apiaries in which colonies were spaced 100 m apart contained lower average mite numbers than 0 or 10 m apiaries. There were interactions among colony type, distance, and sampling date; however, when there were significant differences, mite numbers were always lower in 100 m apiaries than 10 m apiaries. These findings pose the possibility that *Varroa* populations are resource regulated at a landscape scale: near-neighbor colonies constitute reproductive resource for mites in the form of additional bee brood.

## Introduction

*Varroa destructor* is the most damaging parasite of the European honey bee (*Apis mellifera* L.) in the world today (Rosenkranz et al. 2010). A critical regulation point of this and any host-parasite relationship is inter-host transmission, which occurs either vertically from parents to offspring or horizontally between individuals within a population. In the context of a honey bee colony, we presume for our present purposes that evolution is acting primarily at the colony level (Wilson and Sober 1989; Queller and Strassmann 1998) which means that horizontal transmission is best understood as action occurring between colonies, not between individuals within a colony. Therefore, horizontal transmission in the *A. mellifera*/*V. destructor* system occurs through adult bee drifting and robbing (Sakofski and Koeniger 1988; Sakofski et al. 1990).

Drifting results when a honey bee leaves one colony and joins another (Free 1958). This phenomenon is common in managed apiaries where honey bee colonies are often placed in rows and in close proximity to each other. In managed situations, drifting is affected by hive arrangement, inter-colony distance, distance from windbreaks, presence of landmarks, direction of colony entrance, topography, and hive color (Jay 1965, 1966a, b, 1968). Drifting is ultimately caused by homing errors made as foraging honey bees return to the colony (Free 1958); however, Forfert et al. (2015) showed that colonies with high mite infestation had significantly enhanced acceptance of drifters. They postulate that the increase in drifter acceptance is attributed to an impaired ability for guard bees to assess incoming heterocolonial foragers. It has been shown in numerous studies that developing honey bees parasitized by mites are less involved in brood care, hive ventilation, and food collecting (Annoscia et al. 2015), and show reduced homing abilities (Kralj and Fuchs 2006). A model calculated by Pfeiffer and Crailsheim (1998) predicted that hives placed linearly 26 cm apart and facing the same direction contain up to 42 ± 6 % alien workers.

High drifting rates lead to high mite transmission rates; reinfestation rates as high as 75.6 mites/colony/day have been recorded in initially mite-free colonies whose nearest neighbor infested colonies were 200 m distant (Greatti et al. 1992). When Sakofski et al. (1990) monitored weekly immigration of mites throughout a season, they found no difference in mite migration when colonies were placed within a row of infested nearest neighbors or when colonies were placed 10 m away from infested neighbors.

Frey and Rosenkranz (2014) found that colonies located in areas with high colony density (>300 colonies within flight range of test colonies) had significantly higher mite invasion over a 3.5-month period compared to colonies in a low density area (50 small nucleus colonies treated for mites before the study). Immigration rate in high density colonies averaged 462 ± 74 mites per colony over the 3.5-month period, while low density colonies received 126 ± 16 mites.

Mite reinfestation and subsequent population increase were attributed to an increase in honey bee colony density by Seeley and Smith (2015). Colonies in their study consisted of 24 hives painted the same color, with entrances facing the same direction, and placed ∼1 m apart in high-density apiaries or 21–73 m apart in low-density apiaries. Colonies that swarmed in low-density apiaries had lower mite numbers and were able to maintain low mite levels, leading to an increase in winter survival. Colonies in high-density apiaries showed a reduction in mites immediately after swarming; however, mite numbers quickly rebounded, leading to increased winter mortality. This rebound in mite population in high-density colonies was attributed to an influx of mites via drifting and robbing from non-swarming colonies within the apiary. The high-density apiary was found to have significantly more drone drift than the low-density apiary. This marked increase in drone drift is a potential explanation for the rapid transmission of mites among colonies in the high-density apiary.

Frey et al. (2011) found no significant difference in number of mites transferred from heavily infested colonies into colonies located at distances of 1, 30, 400, 1300, or 1500 m. The number of invading mites per colony raged from 85 to 444 mites within the 2-month test period. The authors noted that during the testing period, there was little forage available, and therefore colonies at all distances potentially robbed weakened and collapsed, heavily-infested colonies. This might explain the relatively equal number of transferred mites observed over varying distances.

Horizontal transmission of mites is known to occur through robbing and drifting, even at great distances, and an increase in colony numbers within the flight range of any one colony amplifies the number of invading mites. Epidemiological theory predicts that a parasite’s virulence evolves to higher levels in populations with higher levels of horizontal transfer of the parasite (Bull 1994; Nowak and May 1994). In the *V. destructor*/*A. mellifera* relationship, increasing mite populations, whether by horizontal transmission (immigration) or endemic growth, are associated with increasing host colony morbidity and eventual death (Harbo 1996; Delaplane and Hood 1999; Seeley and Smith 2015). Therefore, it is important to explore the effects of colony distance on horizontal transmission of *Varroa*, not only because closer distances increase immigration and lead to greater populations of mites and greater colony morbidity, but also because increases in host population densities are predicted to select for more virulent strains of parasites.

**Figure 1.**
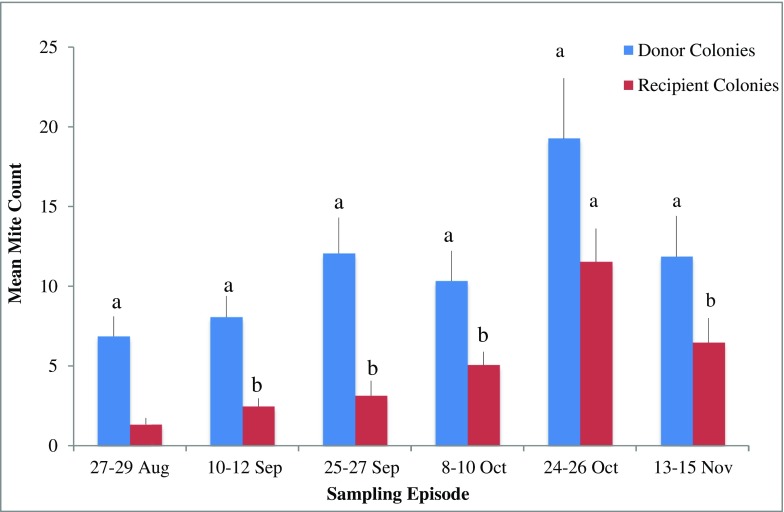
Interaction between colony donor type (mite-donor or -recipient) and sampling episode. Episodes before donor colonies were inoculated with mites are omitted. *Different letters* indicate significant differences between colony type within sampling episode. *Error bars* represent SE of the least squares means separation.

Owing to a long history of beekeeping, there are two ways to think about inter-colony distance in the context of mite transmission and virulence in *A. mellifera*: that existing in natural unmanaged bee populations and that encountered in managed apiaries. Average inter-colony distances in nature range from 304 to 4848 m (mean = 2326 ± SD = 1031, *n* = 45; derived from Figure 1, Seeley et al. 2015), whereas distances in apiaries are smaller by orders of magnitude; inter-colony distances of 1 m are not uncommon. With a range of possibilities this wide, we decided to focus on and replicate inter-colony distance to nearest neighbor as a driver in mite emigration and population growth.

In the present study, we placed mite-free colonies at distances of 0, 10, or 100 m from a nearest neighbor mite-infested colony and monitored mite levels and subsequent colony strength over a season. Our design differs from others because it replicates inter-colony distance and standardizes nearest neighbor condition while approaching inter-colony distances realistic for both natural and managed situations.

## Materials and methods

The study utilized 15 apiaries, each comprised of two honey bee colonies. Each apiary pair was randomly assigned one of three inter-colony distances: 0, 10, or 100 m (5 apiaries each distance). Apiaries were located at least 3.2 km from each other or any other known honey bee colony; all were within 24 km of Athens, Georgia, USA (33.9500°N, 83.3833°W). Hives within each apiary were painted the same color and were faced in the same cardinal direction, at the same elevation, to normalize drifting propensity within the apiary.

Colonies were started on 14–15 Jun 2012 from three-pound (1.4 kg) packages and mated queens purchased from the same supplier. All packages were rendered nearly mite-free by treatment with 2.8 % oxalic acid solution, applied at the rate of 3.0 mL solution per 1000 bees, and using the protocol of Aliano and Ellis (2009), which is expected to reduce mite levels by >90 %. Treatment was given 3 days after packages were made, and bees remained in packages 3 days post-treatment. Packages were housed in standard 10-frame Langstroth hives with screen bottom boards. Each colony was given two drawn combs and eight undrawn waxed plastic frames. Honey supers were added mid-summer to accommodate incoming nectar. Queen excluders were used, and colonies were fed 1:1 sugar water mixture as needed.

One colony in each apiary pair was randomly selected to receive 300 mites (donor colony). Inoculations were carried out 31 Jul–9 Aug. Live mites were collected from off-site, heavily-infested colonies by dusting top bars with powdered sugar and collecting mites that fell through screen bottom boards onto a white piece of corrugated plastic. Mites from multiple colonies were collected in this fashion, pooled together in the field, brought back to the lab, and counted into 300-mite cohorts. Mites were gently washed under lukewarm water to remove sugar, transferred onto moistened filter paper, and kept in an incubator at 32 °C and ∼40 % relative humidity until inoculation. All mite inoculations were performed the same day as mite collection and were carried out by removing a brood frame from the target colony, brushing off adult bees, laying the frame horizontally across the hive, and gently pouring 300 mites onto an area of open brood. The frame was left in this position until mites were able to enter brood cells or hold onto cells. The frame was then carefully returned to the colony.

Relative mite counts were made using sticky screen counts on bottom boards on 14–19 Jun, 19–22 Jun, 29 Jun–2 Jul, 13–17 Jul, 27–29 Aug. 10–12 Sep, 25–27 Sep, 8–10 Oct. 24–26 Oct, and 13–15 Nov. Only sampling episodes from 27–29 Aug through 13–15 Nov were included in statistical analyses, since donor colonies were not inoculated until 31 Jul-9 Aug; however, sampling prior to donor inoculation was done to ensure that colonies were as free of mites as possible. Baseline mean mites collected over all colonies for the first sampling episode, 14–19 Jun, was 167.6 ± 31.2 (mean ± SE). The means had reduced to 4.1 ± 1.3 by the subsequent episode, 19–22 Jun. A treatment using the miticide Amitraz was administered after the 19–22 Jun sampling episode to further lower incipient mite levels. On the subsequent two sampling episodes, 29 Jun–2 Jul and 13–17 Jul, mean mite numbers had dropped to 1.4 ± 0.4 and 0.1 ± 0.1, respectively. On the 13–17 Jul sampling episode, no colony had more than one mite on a sticky screen count. In addition to relative sticky screen counts, total mite populations were determined at the start and conclusion of the study. Obtaining total mite populations required determining number of mites in brood and summing with phoretic mites on adult bees. Mites in brood were estimated by uncapping 100 worker bee brood cells and inspecting for mites. Phoretic mites were assessed using the alcohol wash method (∼300 adult bees) (Dietemann et al. (2013).

Total adult bee population, capped worker brood, and capped honey were estimated following section 4.2 in Delaplane et al. (2013). By knowing total adult bees and total capped brood, we were able to estimate colony mite populations. The ratio of mites in brood to total mites in each colony was determined as a proxy measure of fecundity of the mite population as described by Harbo (1996).

Analyses of sticky screen counts were conducted using the mixed model GLIMMIX procedure, SAS Institute 1992, recognizing inter-colony distance (0, 10, or 100 m), colony type (mite-donor or -recipient), and sampling episode as fixed effects and apiary replication as random effect. The data were analyzed using a GLIMMIX model coded for a Poisson distribution to account for conditional residuals showing skewness in the data. Tests were run for all two- and three-way interactions among fixed effects. Model means are reported for distance, colony type, and relevant interactions, but multiple comparisons (using Holm-Tukey) were run on least squares means.

The colony strength analysis also used the mixed model GLIMMIX procedure, SAS Institute 1992, recognizing inter-colony distance and colony type as fixed effects. In the case of bee population and capped brood cells, the initial values for these parameters at start-up were included as covariates but later discarded when they failed to explain any variation in the models.

## Results

**Figure 2.**
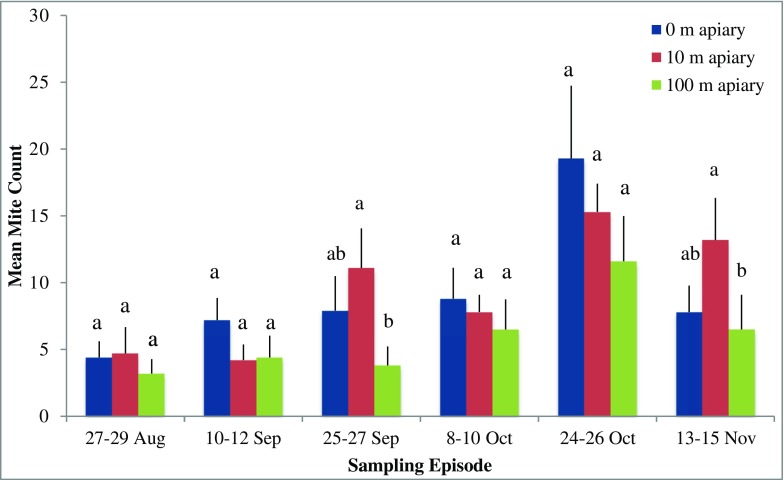
Interaction between apiary inter-colony distance and sampling episode. Episodes before donor colonies were inoculated with mites are omitted. *Different letters* indicate significant differences among colony distances within sampling episode. *Error bars* represent SE of the least squares means separation.

**Figure 3.**
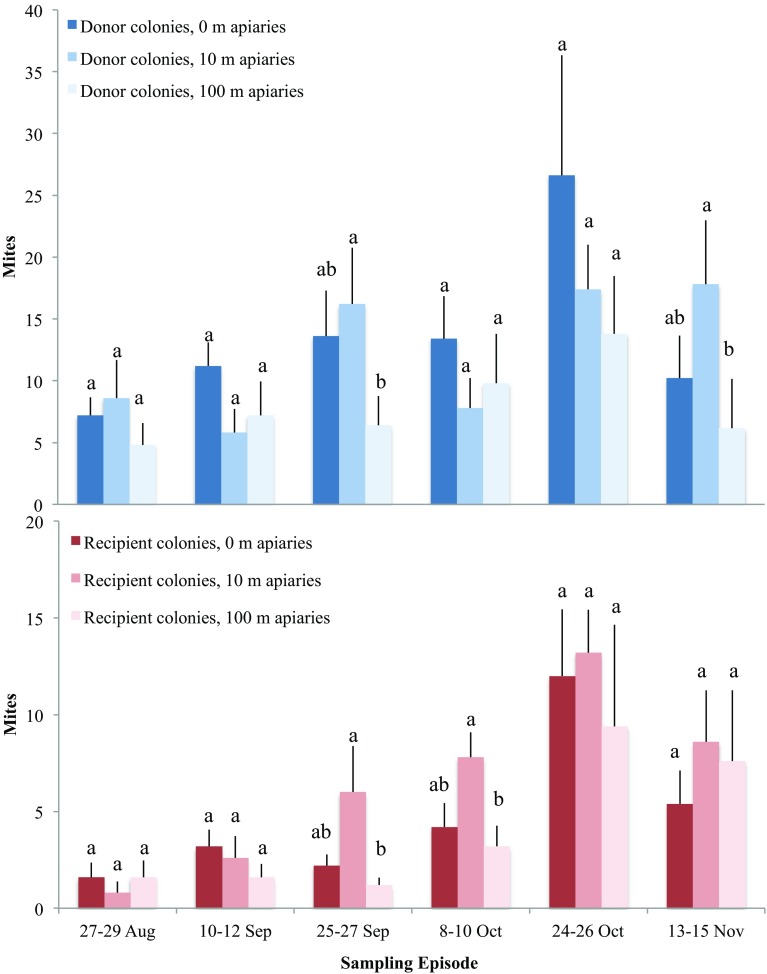
Interaction between colony distance, colony donor type (mite-donor or -recipient), and sampling episode. Episodes before donor colonies were inoculated with mites are omitted. *Different letters* indicate significant differences among colony distances within colony donor type (mite-donor or -recipient) and sampling episode. *Error bars* represent SE of the least squares means separation.

Sticky screen mite drop counts were significantly affected by all three main effects: apiary inter-colony distance (*F* = 3.8, df = 2, 12, *P* = 0.05), colony type (mite-donor or -recipient) (*F* = 17.6, df = 1, 12, *P* = 0.001), and sampling episode (*F* = 54.0, df = 5120, *P* < 0.0001, Table I). Additionally, there were significant interactions between colony type*sampling episode (*F* = 8.2, df = 5120, *P* < 0.0001, Figure 1), distance*sampling episode (*F* = 3.6, df = 10,120, *P* = 0.0003, Figure 2), and colony type*distance*sampling episode (*F* = 2.1, df = 10,120, *P* = 0.03, Figure 3).

**Table I:** Model means (± SE) for mite sticky screen drop counts pooled by sampling episode over all distances and mite-donor/-recipient colonies.

27–29 Aug	10–12 Sep	25–27 Sep	8–10 Oct	24–26 Oct	13–15 Nov
2.6 ± 0.4a	3.8 ± 0.5b	4.7 ± 0.6b	6.1 ± 0.7c	12.9 ± 1.3d	7.5 ± 0.8c

Counts with different letters are significantly different at *P* < 0.001. Analyses (see text) were run on least squares means. In all cases, *n* = 30

When pooled by apiary inter-colony distance, the whole model analysis showed that mean mite counts were separated by Holm Tukey in the following pattern: 100 m apiaries <(0 or 10 m apiaries) with means of 6.0 ± 0.9 (mean ± SE), *n* = 60 in 100 m apiaries; 9.4 ± 1.0, *n* = 60 in 10 m apiaries; and 9.2 ± 1.3 *n* = 60 in 0 m apiaries.

When pooled by colony type, mite-donor colonies had significantly more mites 11.4 ± 1.0 *n* = 90 than recipient colonies 5.0 ± 0.6 *n* = 90.

Table I shows model means for sticky screen counts pooled by sampling episode. The data shows that mite populations significantly increased over the study period and then moved downward on the last sampling episode, a pattern typical of mite populations as winter approaches and honey bee brood production contracts.

Figure 1 shows the interaction of colony type and sampling episode. Donor colonies had significantly higher sticky screen counts than recipient colonies on all episodes except 24–26 Oct.

Figure 2 shows the interaction between apiary inter-colony distance and sampling episode. Mean mite counts on sticky screens were significantly higher in 10 m apiaries compared to 100 m apiaries on both the 25–27 Sep and 13–15 Nov sampling episodes. Otherwise, there were no differences by distance on other episodes, nor did the patterns necessarily match 25–27 Sep or 13–15 Nov.

Figure 3 shows interactions among colony type*distance*sampling episode.

For donor colonies, mean mite counts were significantly higher in the 10 m apiaries compared to 100 m apiaries on the 25–27 Sep and 13–15 Nov sampling episodes. For recipient colonies, mean mite counts were significantly higher in 10 m apiaries compared to 100 m apiaries on the 25–27 Sep and 8–10 Oct sampling episodes. There were no differences among distances by colony type on other sampling episodes, nor did the patterns necessarily match those episodes in which differences occurred.

Analyses of ending strength parameters found no significant effects of distance and colony type on adult bee populations, capped brood cells, total mites per colony, nor percent mites in brood (*P* ≥ 0.05). Nevertheless, natural means and *n* for each strength parameter are provided in Table II, grouped by apiary inter-colony distance.

**Table II:** Natural means (± SE) for adult bee populations, capped brood cells, total mites per colony, and percent mites in brood. In all cases *n* = 10.

	0 m apiaries	10 m apiaries	100 m apiaries
Adult bees	7504 ± 802	7956 ± 719	8855 ± 1139
Capped brood	669 ± 479	786 ± 307	1288 ± 557
Total mite population	319 ± 63	589 ± 114	453 ± 118
Percent mites in brood	7 % ± 5	10 % ± 5	14 % ± 7

## Discussion

Our results add to a growing base of evidence that spatial structure of honey bee communities, in particular inter-colony distance, significantly affects colony *Varroa* mite numbers. By varying and replicating inter-colony distance over multiple apiaries, we were able to detect effects of nearest neighbor mite-source colony on mite transmission to uninfested colonies. Relative mite numbers were measured over a 4-month period in order to observe and compare changes in mite levels at different distances over time.

Mite numbers increased steadily from the 27–29 Aug through 24–26 Oct sampling episodes and then showed a significant decrease in the 13–15 Nov episode (Table I). This result is a predictable outcome of the fact that mite population growth is regulated in part by seasonal availability of bee brood (Fries et al. 1994; Calis et al. 1999; Vetharaniam 2012).

The trend for mite increase was similar in both donor and recipient colonies, with recipient colonies being significantly and predictably lower in all but one sampling episode (Figure 1). When pooled, donor colonies had significantly more mites than recipient colonies (see section 3).

We cannot discriminate whether mite growth over time was caused by drift, endemic mite reproduction, or a combination of the two, but finding overall mite levels significantly lower in the 100 m apiaries compared to 10 m apiaries suggests that drifting plays a significant part. This interpretation is supported by previous studies showing that increases in horizontal transmission directly correlate to increases in drift brought about by higher colony densities and smaller inter-colony distances (Sakofski and Koeniger 1988; Sakofski et al. 1990; Sakofski 1991; Frey et al. 2011; Seeley and Smith 2015). On the interaction analysis (Figure 2), apiaries grouped by sampling episode differed depending on distance. However, on dates where significant differences were observed, the pattern was always 100 m apiaries having fewer mites than 10 m apiaries, with 0 m apiaries intermediate. It appears that the 0 and 10 m inter-colony distances were biologically indistinguishable with regard to bee drift and mite transmission, and we cannot offer a biological speculation why the 0 m apiaries interacted as intermediates.

The consistency of 100 m apiaries having fewer mites than 10 m apiaries is sustained in the three-way interaction of distance*colony donor type*sampling episode (Figure 3). The continuity of lower mite numbers in the 100 m apiaries suggests a biologically meaningful threshold. This finding is consistent with that for the comparatively large inter-colony distances found in nature (range = 304–4848 m, see section 1, Seeley et al. 2015).

We expected effects on mite numbers across recipient colonies at different inter-colony distances due to varying rates of horizontal mite transmission; however, variation across donor colonies was unexpected. Furthermore, donor colonies followed similar mite progression patterns observed for recipient colonies (Figure 3). One hypothesis that explains higher mite numbers in donor colonies in more closely spaced apiaries posits that competition for larval hosts is less keen in those apiaries than in apiaries with colonies 100 m from their nearest neighbor. An increase in horizontal transmission enabled mites to more quickly exploit brood of the nearby and relatively mite-free recipient colonies. Indeed, an important component regulating colony mite population growth is availability of honey bee brood (Calis et al. 1999). Our findings suggest the possibility that this intra-colony mite population model can be expanded to the level of colony community at a landscape scale.

As shown in Table II, we found no effects of apiary inter-colony distance on numerous proxy measures of colony fitness. This result is likely an artifact of the relatively short time scale of the study; honey bee colonies with low initial mite populations do not show deleterious effects of mite infestation in temperate latitudes such as Georgia, USA, until at least two seasons of unregulated growth (Calis et al. 1999). By utilizing colonies that were virtually mite-free at the onset, even donor colonies seeded with 300 adult female mites failed to reach the economic treatment threshold of 59–187 mites per 24 h sticky screen drop established by Delaplane and Hood (1999) for the American Southeast and later confirmed by Delaplane et al. (2010).

However, the close association between mite population growth and increasing colony morbidity is firmly established (Harbo 1996; Delaplane and Hood 1999; Delaplane et al. 2010), and the decrease in apiary-level counts of parasitic Varroa mites we detected at increasing inter-colony distances is consistent with epidemiological theory that predicts decrease in parasite transmission and virulence at decreasing host densities (Bull 1994; Nowak and May 1994; Lipsitch et al. 1996; Fries and Camazine 2001; Schmid-Hempel 2011). Furthermore, crowding of colonies within apiaries (Seeley and Smith 2015) and crowding of apiaries within landscapes (Frey and Rosenkranz 2014) have been independently shown to increase mite transmission. The current study builds upon these and other studies by replicating inter-colony distance and detecting evidence of Varroa population regulation by brood availability at the level of landscape.
